# Nonmyeloablative Allogeneic Stem-Cell Transplantation for Metastatic Renal Cell Cancer: A Review and Update

**DOI:** 10.1100/tsw.2006.391

**Published:** 2006-12-15

**Authors:** P. Erotocritou, H. Ahmed, S.S. Patel, I. Shergill, H.R. Patel, P.M. Shah, M. Arya

**Affiliations:** The Institute of Urology and Nephrology, University College London, U.K. and The Gujarat Cancer and Research Institute, MP Shah Cancer Hospital, Ahmedabad, India

**Keywords:** allogeneic stem cell, metastatic renal cell cancer, myeloablative, nonmyeloablative, immunotherapy

## Abstract

Metastatic renal cell carcinoma (RCC) is resistant to conventional chemotherapy and radiotherapy. However, immunotherapy appears to be effective in 15—20% of cases, with interleukin-2 becoming the standard therapy for this disease. As a consequence of the immune susceptibility of RCC, other avenues of immunotherapy are being explored, such as nonmyeloablative allogeneic stem cell transplantation (NST). A number of trials have shown NST to be effective in varying degrees, causing partial or complete regression. Although nonmyeloablative conditioning is safer than myeloablative conditioning, its role has yet to be clearly proven as many studies have shown variable effect. Alongside this limitation, transplant-related toxicity also forms obstacles. Regardless of the limitation of NST, further refinement of the technique, with appropriate patient selection, may lead to this being an effective therapeutic choice for a significant number of individuals.

## INTRODUCTION

An estimated 6600 people will be diagnosed with renal cell carcinoma (RCC) in the U.K. every year, representing 2% of all cancer diagnoses, with approximately 3600 cancer-specific deaths. RCC typically occurs between the 6$^{th}$ and 7$^{th}$ decades and affects men almost twice as often as women[1].

The classical presentation of RCC with loin pain, haematuria and a renal mass somewhat belies the fact that this triad is only present in 5% of cases. It is estimated that between 50–60% of RCC cases are identified as incidental lesions during a radiological examination[2,3]. In the modern era, an increasing number of patients are diagnosed at an earlier stage due to the increased use of diagnostic testing with ultrasound, computed topography, or magnetic resonance imaging. However, many still have advanced disease at presentation; a quarter of patients present with advanced disease that is either locally invasive or metastatic whilst a third of patients with localised disease will subsequently have a recurrence[4]. Metastatic disease has a poor prognosis with a median survival of 6–10 months and an overall 2-year survival of 10–20%[5]. Metastatic RCC (mRCC) is a treatment challenge with low response rates to many chemotherapeutic agents. Therefore, the need to identify new therapeutic agents and novel therapeutic approaches is paramount[6].

In this article, we review the reasons why traditional chemoimmunotherapeutic agents have limited effect on mRCC, and discuss the rationale and the results to date for using allogeneic stem cell transplantation in this aggressive disease.

## REVIEW CRITERIA

A comprehensive search of Medline, PubMed, and Current Contents Databases was carried out for publications between January 1980 and August 2006 using the search terms 'renal carcinoma or renal cancer' in combination with 'allogeneic stem cell transplantation' or 'immunotherapy' or 'chemotherapy'. Manual searches of articles to supplement electronic searches were also carried out.

## CHEMOTHERAPY FOR RENAL CELL CARCINOMA

Conventional chemotherapy-based treatment of metastatic disease has response rates of less than 15% and clearly fails to prolong survival[7]. Recently, a number of studies have investigated the use of single agent and a combination of agents (which have been shown to be successful in treating other cancers) in the treatment of mRCC[8,9,10,11,12]. It was disappointing that none of these studies demonstrated encouraging effects beyond the response rates of 15% or less shown by Yagoda et al. in 1993. It has been postulated that one important reason might be the overexpression of the multidrug-resistance (MDR) genes/P-glycoprotein 170, which act as efflux pumps that reduce the intracellular concentration of drugs and hence their effectiveness.

## IMMUNOTHERAPY FOR RENAL CELL CARCINOMA

The immune system has an important role in the control and potential treatment of mRCC. In 1928, Bumpus reported the first case of spontaneous regression of mRCC postnephrectomy, which was attributed to an antibody-mediated immune response[13]. The incidence of spontaneous regression is thought to be less than 1%[14] and RCC has been found to undergo spontaneous regression more frequently than other cancers[15]. The mechanism whereby this occurs is poorly understood, but thought to be immune related.

The two cytokines explored for mRCC immunotherapy are interleukin-2 (IL-2) and interferon alpha (IFN-α). IL-2 is involved in the growth and differentiation of T cells, B cells, and natural killer cells. Thus, it does not have a direct antitumour activity, but is thought to modify and up-regulate tumour-host immune responses. This lymphokine has demonstrated a clinical response rate of 10–20% in patients with mRCC[16], which is only slightly greater than chemotherapy. However, in one study, 5–7% of patients had a complete response that lasted for 2 years in 80% of these patients[17]. In two recent randomised studies, high-dose IL-2 led to a higher complete response rate than low-dose IL-2[18,19]. Worryingly, IL-2 treatment is associated with a mortality of approximately 1%, although this value is decreasing[17].

INF-α is involved in the activation of natural killer (NK) cells and macrophages[20], and has a clear role as an antitumour agent[21]. Antiangiogenic effects of IFN-α have also been observed[22]. Response to IFN-α is approximately 12%[23], although greater survival is associated with a high-performance status of the patient and those who are treated postnephrectomy[24]. Interestingly, a combined complete and partial response of 30% has been reported[25]. Long-term survivors have been seen with IFN-α, although duration of response rarely exceeds 2 years[24]. Combined treatment with IFN-α and IL-2 can increase response rates, but not survival; this was demonstrated by a response rate at 25 weeks of 2.9% with Il-2, 6.1% with INF-α alone, and 13.6% with the two combined[26]. Although both these agents have clearly helped to reduce the disease burden in a significant number of sufferers, there is still much room for improvement and long-term survival of mRCC treated with cytokines remains a rare event[27].

## CHEMOIMMUNOTHERAPY FOR RENAL CELL CARCINOMA

The combination of cytokine immunotherapy with chemotherapeutic agents can potentially further enhance antitumour activity. Regimens combining immunotherapy with intravenous (IV) 5-fluorouracil (5-FU) achieved response rates varying from 12–39%[28,29,30], whilst combinations with IV vinblastine gave response rates between 20–39%[30,31,32]. 13-cis-Retinoic acid (po-13Cra) given orally is another agent that may be added to regimens as it is involved in regulation of cell differentiation. It enhances antitumour efficacy of INF-α[33], as well as combined INF-α and IL-2 therapy with response rates varying between 17–30%[34,35]. Atzpodien et al.[36] have recently compared groups of patients on four differing regimens. They showed objective response rates of 29% with subcutaneous (sc) IL-2, sc-INF-α-2a, and po-13Cra. When either inhaled IL-2 or IV 5-fluorouracil or po-Capecitabine were added to this regimen response rates of 31, 19, and 26% were obtained respectively. This trial did not show any significant beneficial effect in adding to the sc-IL-2/sc-INF-α-2a/po-13cra regimen. Another recently published trial of low-dose vinorelbine combined with IL-2 showed a response rate of 43%. Whilst this is promising, the numbers involved were too small for definitive conclusions to be made with regard to this treatment[37].

It is also necessary to briefly mention some other biological treatments that have shown some promise recently. A number of review articles are available on the subject[38,39,40,41,42]. Detailed discussion of these agents is beyond the scope of this review, but suffice to say that the majority of these agents are targeted at vascular endothelial growth factor (VEGF) and its signalling pathways. Phase II studies have shown promising activity for these agents and results of phase III studies will determine the role of these agents in mRCC.,

In fact, many of the trials to date with chemoimmunotherapy do not provide definitive information on treatment and, as a consequence, other approaches, such as allogeneic stem cell transplantation, have been explored as an alternative therapeutic modality.

## ALLOGENEIC STEM CELL TRANSPLANTATION

This procedure has been used successfully to treat patients with haematological malignancies for many years. It involves intensive chemotherapy or radiotherapy in order to induce a direct cytotoxic effect on malignant cells whilst ensuring that the stem-cell allograft is not rejected. This is followed by infusion of human-leukocyte-antigen (HLA)–compatible donor haematopoietic stem cells from bone marrow or peripheral blood stem cells. This allows the recipient to restore haematopoietic function and to replace NK cells and T and B lymphocytes. These donor immune cells mediate potent antitumour effects on malignant cells that survive the conditioning regimen[43]. Such a donor immune–mediated antitumour response is termed graft-versus leukaemia or graft-versus tumour (GVT), and is recognised to have a curative capacity independent of the cytotoxic conditioning regimen. Immunosuppressive agents that act as prophylaxis against graft-versus-host-disease (GVHD) are usually withdrawn after 30–60 days, provided GVHD is not active.

Initial evidence supporting the GVT effect came from animal studies showing that allogeneic T cells could induce a GVT effect in murine mammary carcinoma[44]. Evidence of GVT effects in solid tumours in humans came from patients with metastatic breast cancer who were observed to have partial regression of metastatic disease during periods of acute GVHD. Eibl et al.[45] reported the case of a 55-year-old woman suffering from refractory breast cancer who underwent stem cell transplantation from an HLA

identical sibling and showed regression of a liver metastasis coinciding with the onset of acute GVHD. Ueno et al.[46] published a series of ten patients with metastatic breast cancer who were treated with stem cell transplantation. They showed 6 out of 10 patients with an objective remission. However, failure of patients to achieve complete responses and the regimen-related mortality (25–30%) associated with myeloablative conditioning hindered further investigations. As myeloablative conditioning is associated with marked mortality and because RCC is refractory to chemotherapy, a nonmyeloablative approach offered potential for reduced toxicity whilst maintaining or approaching the cancer control effect of myeloablative techniques. Therefore, this approach seemed the correct direction to take in investigating the role of allogeneic stem cell transplantation in mRCC.

## NONMYELOABLATIVE ALLOGENEIC STEM CELL TRANSPLANTATION (NST)

This procedure relies on sufficient immunoablation of the host by reduced intensity conditioning (RIC) before NST, but without causing myeloablation. It is therefore suitable in those patients who are unfit or would stand a high risk of mortality or substantial morbidity from myeloablative regimens. It is still necessary to institute immunosuppression after NST to avoid graft rejection and early GVHD[47].

What is interesting about this treatment is the phenomenon of mixed chimerism found in patients post-transplantation in which recipient and donor cells coexist in the peripheral blood. This is advantageous as it aids engraftment and decreases GVHD, but unfortunately also reduces the GVT effect. A substantial GVT effect is only seen when complete donor T-cell chimerism takes place, usually after withdrawal of post-transplantation immunosuppression or when post-transplant donor lymphocyte infusion (DLI) is given. NST has been shown to be effective in haematological malignancies[48,49] and is now being studied in a range of solid tumours including mRCC.

## NONMYELOABLATIVE ALLOGENEIC STEM CELL TRANSPLANTATION IN METASTATIC RENAL CELL CANCER

The first clinical data on the treatment of mRCC with NST was published by Childs et al. in 2000[50]. In this study, 19 patients who were refractory to immunotherapy underwent NST from a related HLA-compatible donor. A sustained engraftment of the allograft was achieved in all patients. In addition, eight patients received DLI for conversion of mixed chimerism to complete donor T-cell chimerism or if rapid tumour progression occurred. Seven of these patients had a partial response and three had a complete response. The responses were typically delayed, and in most responding patients, this only occurred after immunosuppression withdrawal and/or when DLI was instituted. Again, tumour response was correlated with the onset of GVHD supporting GVT as the primary mechanism of action. In this series, two patients died of transplant-related complications.

Since this initial series, a number of other groups have evaluated NST for mRCC, using various conditioning regimens and GVHD prophylactic agents (Table 1). The response rates of these trials has varied from 8–57% as has the transplant related mortality (TRM), with two of these trials showing no tumour response at all (Table 2).

Two series showed no GVT effect at all and, interestingly, these showed that patients died earlier due to tumour progression at a median of 3 and 5.5 months[53,60]. The fact that GVT effect is delayed for solid tumours whilst tumour progression may be promoted during GVHD prophylaxis[63] points to the importance of appropriate patient selection. In addition, the response rate variation across series, even when similar regimens were used, further suggests that patient selection may account for those series with higher response rates[64]. Which group of mRCC patients would benefit from NST is presently difficult to evaluate. It may be that selecting patients who are refractory to immunotherapy may in itself lead to a more aggressive group being treated in these studies and diminish treatment effect (see Table 1).

However, using NST as a primary measure in mRCC in future phase II trials may not be justifiable ethically since the treatment carries such high risk to the patient.

**TABLE 1**
**Previous Failed Immunotherapy (%), Nonmyeloablative Conditioning Regimens, and GVHD Prophylaxis**

| Study | Previous Failed Immunotherapy (%) | Conditioning Regimen | GVHD Prophylaxis |
|---|---|---|---|
| Childs et al.[50] | 89 | Cyclophosphamide<br>Fludarabine | Cyclosporine |
| Rini et al.[51] | 61 | Cyclophosphamide<br>Fludarabine | Tacrolimus<br>Mycophenolate mofetil (MMF) |
| Bregni et al.[52] | 100 | Cyclophosphamide<br>Fludarabine<br>Thiopeta | Cyclosporine<br>Methotrexate |
| Pedrazzoli et al.[53] | 100 | Cyclophosphamide<br>Fludarabine | Cyclosporine<br>Methotrexate |
| Hentschke et al.[54] | 30 | Fludarabine, 2 Gy total<br>Body irradiation (#) | MMF<br>Cyclosporine |
| Ueno et al.[55] | 80 | Fludarabine<br>Melphalan | Tacrolimus<br>Methotrexate |
| Nakagawa et al.[56] | 100 | Cladarabine or<br>Fludarabine (*)<br>Busulfan<br>Antithymocyte globulin(ATG) | Cyclosporine |
| Massenkeil et al.[57] | 100 | Cyclophosphamide<br>Fludarabine<br>ATG | Cyclosporine (+) |
| Blaise et al.[58] | Not documented | Fludarabine<br>Busulfan<br>ATG | Cyclosporine |
| Tykodi et al.[59] | 75 | Fludarabine, low dose<br>Total body irradiation | Cyclosporine<br>MMF |
| Rini et al.[60] | 100 | Fludarabine<br>Cyclophosphamide | Tacrolimus<br>Methotrexate |

\# Recipients of unrelated NST were also given thymoglobulin and 2 additional days of fludarabine (five patients).

* First three received cladarabine and remainder received fludarabine as cladarabine was no longer available.

\+ One patient received MMF in addition after an unrelated donor transplantation.

Failed immunotherapy was either IL-2, INF-α, or both.

## COMPLICATIONS OF NONMYELOABLATIVE ALLOGENEIC STEM CELL TRANSPLANTATION

Despite the reduced toxicity of low-intensity conditioning, the mortality rate is substantial in a population of patients that generally has a poor performance before treatment because of malignant disease. The mortality from conditioning regimens in HLA-matched donors is still 10–20%, and greater than 20% if unmatched donor stem cells are used.

With the regimens used in stem cell transplantation, there can be a number of complications, such as infections (bacterial, fungal, and viral), venoocclusive liver disease, haemorrhagic cystitis, and most importantly, GVHD. These complications present barriers to safe and effective use of this treatment method.

**TABLE 2**
**Transplant Related Mortality (TRM), GVT Response, and Patient Numbers**

| Study | Patient Number | GVT Effect (%) | TRM (%) |
|---|---|---|---|
| Childs et al.[50] (*) | 19 | Yes (53%) | 12 |
| Rini et al.[51] | 18 | Yes (22%) | 14 |
| Bregni et al.[52] (+) | 7 | Yes (57%) | 0 |
| Pedrazzoli et al.[53] | 7 | No | 29 |
| Hentschke et al.[54] | 10 | Yes (30%) | 20 |
| Ueno et al.[55] | 15 | Yes (20%) | 20 |
| Nakagawa et al.[56] | 9 | Yes (11%) | 0 |
| Massenkeil et al.[57] | 6 | Yes (33%) | 0 |
| Blaise et al.[58] | 25 | Yes (8%) | - |
| Tykodi et al.[59] | 8 | Yes (13%) | 0 |
| Rini et al.[60] | 22 | No | 9 |

* Recent update from this group reports responses in 21 of 47 patients[61].

\+ Recent update from this group reports responses in 17 of 61[62].

GVHD can be acute (within 2–3 months of transplantation), chronic (3–6 months after transplantation), or both. Acute GVHD causes diarrhoea, dermatitis, and hepatitis, and is treated with a range of immunosuppressive agents. Chronic GVHD has features in keeping with autoimmune disorders, such as scleroderma, and responds to immunosuppressive agents early in the course.

## DONORS

Where possible, the ideal donor is a sibling with the same two HLA haplotypes as the recipient. An identical twin is the most suitable donor from an immunological point of view, but allogeneic stem cell transplantation between twins (syngeneic transplant) is associated with an increased relapse rate[65,66]. Relapse is presumed to be higher because residual cancer does not present alloantigens to donor cells[67]. In some cases, HLA-identical family members other than siblings can be used. In addition, partially HLA-identical family members can be used with success approaching that of matched siblings.

Unfortunately, only 30% of all patients have a family donor and the use of HLA-matched unrelated donors (MUD) has increased over time[68]. Due to improved matching techniques, transplants with MUD have improved and approach the success rate for those undergoing transplantation from siblings[69]. Hentschke et al.[54] treated ten patients with NST for mRCC, but only five had an HLA-matched sibling donor with the remainder receiving MUD transplants. Graft rejection occurred in only one patient who was actually in the matched sibling group. In addition, whilst grade 2–4 GVHD occurred in 40% of the MUD group, it actually occurred in 60% of the matched sibling group.

Umbilical cord blood (UCB) represents another potential donor source for NST as it is rich in haematopoietic stem cells. Advantages of UCB are its rapid availability and the reduced risk of GVHD due to the deficiency of mature T cells. This fact allows for some degree of mismatch, although drawbacks from such an approach include an increased incidence of graft failure and a slower engraftment, which results in more infections. Recent reports have demonstrated the feasibility of cord blood transplantation, using reduced intensity regimens for adult patients with advanced haematological malignancies[70]. Such an alternative donor approach needs to be investigated for NST in solid tumours in order to potentially allow more people to benefit from this therapy. However, this route is controversial. Much of this revolves around storage of stem cells, the consent needed for the blood; parental consent to test for diseases to allow it to be used; and the fear that parents may have another child in order to cure a sibling are difficult issues which have raised concerns[71].

## CONCLUSION

The lack of effective treatments for mRCC continues despite numerous therapeutic strategies. The ability of NST to introduce a donor-immune system into a patient with mRCC may be one modality that can reduce the mortality from this disease in a proportion of patients. However, the majority of studies evaluating the effectiveness of NST have shown variable responses. Nonetheless, they have proven that clinically significant regression of the tumour can occur. There are clear difficulties to overcome. Early indicators show that patients need to be selected with appropriate mRCC disease features, such as slow progression, in order to allow for the timely delivery of NST. In addition, if NST is to become more commonly used, the antitumour GVT effect needs to be separated from the antirecipient GVHD effect.

## References

1. Cancer Research UK (2006) Statistics (www.cruk.co.uk/aboutcancer/statistics/incidence) Accessed August 2006
2. Jayson, M. and Sanders, H. (1998) Increased incidence of serendipitously discovered renal cell carcinoma. *Urology* **51,** 203–205.
3. Pantuck, A.J., Zisman, A., and Belldegrun, A.S. (2001) The changing natural history of renal cell carcinoma. *J. Urol.* **166,** 1611–1623.
4. Lam, J.S., Belldegrun, A.S., and Pantuck, A.J. (2006) Long-term outcomes of the surgical management of renal cell carcinoma. *World J. Urol.* **24(3),** 255–266.
5. Medical Research Council Renal Cancer Collaborators (1999) Interferon-alpha and survival in metastatic renal carcinoma: early results of a randomised controlled trial. *Lancet* **353,** 14–17.
6. Motzer, R.J. (2003) Renal cell carcinoma: a priority malignancy for development and study of novel therapies. *J. Clin. Oncol.* **21,** 1193–1194.
7. Yagoda, A., Petnylak, D., and Thompson, S. (1993) Cytotoxic chemotherapy for advanced renal cell carcinoma. *Urol. Clin. North Am.* **20**, 303–321.
8. Chaouche, N., Pasturaud, A.L., Kamioner, D., Grandjean, M., Franialte, J., and Tourani, J.M. (2000) Oxaliplatin, 5-fluorouracil and folinic acid (Folfox) in patients with metastatic renal cell carcinoma: results of a pilot study. *Am. J. Clin. Oncol.* **23**, 288–289.
9. Rini, B.I., Vogelzang, N.J., Dumas, M.C., Wade, J.L., Taber, D.A., and Stadler, W.M. (2000) Phase II trial of weekly intravenous gemcitabine with continuous infusion fluorouracil in patients with metastatic renal cell cancer. *J. Clin. Oncol.* **18**, 2419–2426.
10. Pagliaro, L.C., Perez, C.A., Tu, S.M., and Daliani, D.D. (2003) Phase II study of capecitabine single-agent therapy in patients with metastatic renal cell carcinoma. *Proc. Am. Soc. Clin. Oncol.* **22,** 443.
11. Stadler, W.M., Halabi, S., Ernstoff, M.S., Barrier, R., Davila, E., Picus, J., and Small, E.J. (2004) A phase II study of gemcitabine (G) and capecitabine (C) in patients with metastatic renal cell cancer (m RCC): a report of cancer and leukaemia group B #90008. *J. Clin. Oncol.* **22,** 4515.
12. Tannir, N.M., Jonasch, E., McMichael, C., Wang, X., Wooten, L., and Ng, C.S. (2005) A phase II trial of gemcitabine (Gx) in patients with advanced renal cell cancer (m Rcc) previously treated with immunotherapy. *J. Clin. Oncol.* **23,** 402s.
13. Bumpus, H.C. (1928) The apparent disappearance of pulmonary metastasis in a case of hypernephroma following nephrectomy. *J. Urol.* **20,** 185.
14. Snow, R.M. and Schellhammer, P.F. (1982) Spontaneous regression of metastatic renal cell carcinoma. *Urology* **20,** 177–181.
15. Challis, G.B. and Stam, H.J. (1990) The spontaneous regression of cancer. *Acta Oncol.* **29,** 545–550.
16. Motzer, R.J. and Russo, P. (2000) Systemic therapy for renal-cell carcinoma. *J. Urol.* **163,** 408–417.

17. Rosenberg, S.A., Yang, J.C., Topalian, S.L., Schwartzenruber, D.J., Weber, J.S., Parkinson, D.R., Seipp, C.A., Einhorn, J.H., and White, D.E. (1994) Treatment of 283 consecutive patients with metastatic melanoma or renal cell cancer using high-dose bolus interleukin-2. *JAMA* **271,** 907–913.
18. Yang, J.C., Sherry, R.M., Stenberg, S.M., Topalian, S.L., Schwartzenruber, D.J., Hwu, P., Seipp, C.A., Rogers-Freezer, L., Marton, K.E., White, D.E., Lieuher, D.J., Merino, M.J., and Rosenberg, S.A. (2003) Randomised study of high-dose and low-dose interleukin-2 in patients with metastatic renal cancer. *J. Clin. Oncol.* **21,** 3127–3132.
19. McDermott, D., Regan, M.M., Clark, J.L., Flaherty, L.E., Weiss, G.R., Logan, T.F., Kirkwood, J.M., Gordon, M.S., Sosman, J.A., Ernstoff, M.S., Tretter, C.P., Urba, W.J., Smith, J.W., Murgolin, K.A., Mier, J.W., Gollob, J.A., Dutcher, J.P., and Atkins, M.B. (2005) Randomised phase III trial of high-dose interleukin-2 versus subcutaneous IL-2 and interferon in patients with metastatic renal cell carcinoma. *J. Clin. Oncol.* **20,** 133–141.
20. Biron, C.A., Nguyen, K.B., Pien, G.C., Cousens, L.P., and Salazar-Mather, T.P. (1999) Natural killer cells in antiviral defense: function and regulation by innate cytokines. *Annu. Rev. Immunol.* **17,** 189–220.
21. Hancock, B.W., Harris, S., Wheatley, K., and Gore, M. (2000) Adjuvant interferon-alpha in malignant melanoma: current status. *Cancer Treat. Rev.* **26,** 81–89.
22. Albini, A., Marchisone, C., Del Grosso, F., Benelli, R., Mansiello, L., Taccheti, C., Bono, M., Ferrantini, M., Rozera, C., Truini, M., Bellardelli, F., Santi, L., and Noonan, D.M. (2000) Inhibition of angiogenesis and vascular tumour growth by interferon-producing cells: a gene therapy approach. *Am. J. Pathol.* **156**, 1381–1393.
23. Wirth, M.P. (1993) Immunotherapy for metastatic renal cell carcinoma. *Urol. Clin. North Am.* **20,** 283–295.
24. Minasian, L.M., Motzer, R.J., Gluck, L., Mazumdar, M., Vlamis, V., and Krown, S.E. (1993) Interferon-alfa-2a in advanced renal cell carcinoma: treatment results and survival in 159 patients with long-term follow-up. *J. Clin. Oncol.* **11**, 1368–1375.
25. Neidhart, J.A., Anderson, S.A., Harris, J.E., Rinehart, J.J., Laszlo, J., Dexeus, F.H., Einhorn, L.H., Trump, D.L., Benedetto, P.W., and Tuttle, R.L. (1991) Vinblastine fails to improve response of renal cancer to interferon alfa-n1: high response rate in patients with pulmonary metastases. *J. Clin. Oncol.* **9,** 832–836.
26. Negrier, S., Escudier, B., Lasset, C., Douillard, J.Y., Savary, J., Chevreau, C., Ravaud, A., Mercatello, A., Peny, J., Mousseau, M., Philip, T., and Tursz, T. (1998) Recombinant human interleukin-2, recombinant human interferon alfa-2a, or both in metastatic renal-cell carcinoma. Groupe Francais d'Immunotherapie. *N. Engl. J. Med.* **338,** 1272–1278.
27. Atzpodien, J. and Reitz, M. (2005) Metastatis renal carcinoma: long-term survivors treated with s.c interferon-alpha and interleukin-2. *Cancer Biotherm. Radiopharm.* **20(4),** 410–416.
28. van Herpen, C.M., Jansen, R.L., Kruit, W.H., et al. (2000) Immunochemotherapy with interleukin-2, interferon-alpha and 5-fluorouracil for progressive metastatic renal cell carcinoma: a multicenter phase II study-Dutch Immunotherapy Working Party. *Br. J. Cancer* **82,** 772–776.
29. Atzpodien, J., Kirchner, H., Illiger, H.J., et al. (2001) IL-2 in combination with IFN-a and FU versus tamoxifen in metastatic renal cell carcinoma: long-term results of a controlled randomized clinical trial. *Br. J. Cancer* **85,** 1130–1136.
30. Atzpodien, J., Kirchner, H., Jonas, U., Bergmann, L., Schott, H., Heynemann, H., Fornara, P., Loening, S.A., Roigas, J., Muller, S.C., Bodenstein, H., Pomer, S., Matzner, B., Rebmann, U., Oberneder, R., Siebels, M., Wandert, T., Puchberger, T., and Reitz, M. (2004) Interleukin-2- and interferon alfa-2a-based immunochemotherapy in advanced renal cell carcinoma: a prospectively randomized trial of the German Cooperate Renal Carcinoma Chemoimmunotherapy Group (DGCIN). *J. Clin. Oncol.* **22**, 1188–1194.
31. Fossa, S.D., Martnelli, G., Otto, U., et al. (1992) Recombinant interferon alfa-2a with or without vinblastine in metastatic renal cell carcinoma: results of a European multi-center phase III study. *Ann. Oncol.* **3,** 301–305.
32. Pectasides, D., Varthalitis, J., Kostopoulou, M., et al. (1998) An outpatient phase II study of subcutaneous interleukin-2 and interferon-alpha-2b in combination with intravenous vinblastine in metastatic renal cell cancer. *Oncology* **55,** 10–15.
33. Aass, N., De Mulder, P.H.M., Mickisch, G.H.S., Mulders, P., van Oosterom, A.T., van Poppel, H., Fossa, S.D., de Prijck, L., and Sylvester, R.J. (2005) Randomized phase II/III trial of interferon alfa-2a with and without 13-cis-retinoic acid in patients with progressive metastatic renal cell carcinoma: the European Organisation for Research and Treatment of Cancer Genito-Urinary Tract Cancer Group (EORTC 30951) *J. Clin. Oncol.* **23,** 4172–4178.
34. Stadler, W.M., Kuzel, T., Dumas, M., et al. (1998) Multicenter phase II trial of interleukin-2, interferon-alpha and 13-cis-retinoic acid in patients with metastatic renal cell carcinoma. *J. Clin. Oncol.* **16**, 1820–1825.
35. Motzer, R.J., Schwartz, L., Law, T.M., et al. (1995) Interferon alfa-2a and 13-cis-retinoic acid in renal cell carcinoma: antitumour activity in a phase II trial and interactions in vitro. *J. Clin. Oncol.* **13**, 1950–1957.
36. Atzpodien, J., Kirchner, H., Rebmann, U., Soder, M., Gertenbach, U., Siebels, M., Roigas, J., Raschke, R., Salm, S., Schwindl, B., Muller, S.C., Hauser, S., Leiber, C., Huland, E., Heinzer, H., Siemer, S., Metzner, B., Heynemann, H., Fornara, P., and Reitz, M. (2006) Interleukin-2/interferon –a2a/13-retinoic acid-based chemoimmunotherapy in advanced renal cell carcinoma: results of a prospectively randomised trial of the German Cooperative Renal Carcinoma Chemoimmunotherapy Group (DGCIN). *Br. J. Cancer* **95,** 463–469.
37. Mencoboni, M.P., Tredici, S., Varaldo, M., Quierolo, G., Durand, F., Rebella, L., Galbusera, V., Pannacciulli, I.M., and Ghio, R. (2006) Chemoimmunotherapy with low dose vinorelbine and interleukin-2 in treatment of patients with metastatic renal cell carcinoma. *Neoplasma* **53(4),** 333–336.

38. Nathan, P., Chao, D., Brock, C., Savage, P., Harries, M., Gore, M., and Eisen, T. (2006) The place of VEGF inhibition in the current management of renal cell carcinoma. *Br. J. Cancer* **94(9),** 1217–1220.
39. Rini, B. (2006) VEGF-targeted therapy in renal cell carcinoma: active drugs and active choices. *Curr. Oncol. Rep.* **8(2),** 85–89.
40. Schrader, A.J., Varga, Z., Pfoertner, S., Goelden, U., Buer, J., and Hofmann, R. (2006) Treatment targeted at vascular endothelial growth factor: a promising approach to managing metastatic kidney cancer. *BJU Int.* **97(3),** 461–465.
41. Patel, P.H., Chaganti, R.S., and Motzer, R.J. (2006) Targeted therapy for metastatic renal cell carcinoma. *Br. J. Cancer* **94(5),** 614–619.
42. Weiss, R.H. and Lin, P.Y. (2006) Kidney cancer: identification of novel targets for therapy. *Kidney Int.* **69(2),** 224–232.
43. Kolb, H.J., Mittermuller, J., Clemm, C., Holler, E., Ledderose, G., Brehm, G., Heim, M., and Wilmann, S.W. (1990) Donor leukocyte transfusions for treatment of recurrent chronic myelogenous leukaemia in marrow transplant patients. *Blood* **76**, 2462–2465.
44. Morecki, S., Yacovlev, E., Diab, A., and Slavin, S. (1998) Allogenic cell therapy for a murine mammary carcinoma. *Cancer Res.* **58**, 3891–3895.
45. Eibl, B., Schwaighofer, H., Nachbaur, D., Marth, C., Gachter, A., Knapp, R., Bock, G., Gassner, C., Schiller, L., Petersen, F., and Niederwieser, D. (1996) Evidence for a graft-versus-tumour effect in a patient treated with marrow ablative chemotherapy and allogenic bone marrow transplantation for breast cancer. *Blood* **88**, 1501–1508.
46. Ueno, N.T., Rondon, G., Mirza, N.Q., Geisler, D.K., Anderlini, P., Giralt, S.A., Andersson, B.S., Claxton, D.F., Gajewski, J.L., Khouri, I.F., Korbling, M., Mehra, R.C., Przepiorka, D., Rahman, Z., Samuels, B.I., Van Besien, K., Hortobagyi, G.N., and Champlin, R.E. (1998) Allogenic peripheral-blood progenitor-cell transplantation for poor risk patients with metastatic breast cancer. *J. Clin. Oncol.* **16,** 986–993.
47. Childs, R., Clave, E., Contentin, N., Jayasekera, D., Hensel, N., Leitman, S., Read, E.J., Carter, C., Bahceci, E., Young, N.S., and Barrett, A.J. (1999) Engraftment kinetics after nonmyeloablative allogenic peripheral blood stem cell transplantation: full donor T-cell chimerism precedes alloimmune responses. *Blood* **94,** 3234–3241.
48. McSweeney, P.A., Niederwieser, D., Shizuru, J.A., Sandmaier, B.M., Molina, A.J., Maloney, D.G., Chauncey, T.R., Gooley, T.A., Hegenbart, U., Nash, R.A., Radich, J., Wagner, J.L., Minor, S., Applebaum, F.R., Bensinger, W.I., Bryant, E., Flowers, M.E., Georges, C.E., Grumet, F.C., Kiem, H.P., Torok-Storb, B., Yu, C., Blume, K.G., and Storb, R.F. (2001) Haematopoietic cell transplantation in older patients with haematologic malignancies: replacing high dose cytotoxic therapy with graft-versus-tumour effect. *Blood* **97**, 3390–3400.
49. Corradini, P., Tarella, C., Olivieri, A., Gianni, A.M., Voena, C., Zallio, F., Ladetto, M., Falda, M., Lucesole, M., Dodero, A., Ciceri, F., Bendetti, F., Rambaldi, A., Sajeva, M.R., Tresoldi, M., Pileri, A., Bordignon, C., and Bregni, M. (2002) Reduced-intensity conditioning followed by allografting of haematopoietic cells can produce clinical and molecular remissions in patients with poor-risk haematologic malignancies. *Blood* **99,** 75–82.
50. Childs, R., Chernoff, A., Contentin, N., Bahceci, E., Schrump, D., Leitman, S., Read, E.J., Tisdale, J., Dunbar, C., Linehan, W.M., Young, N.S., and Barnett, A.J. (2000) Regression of metastatic renal-cell carcinoma after nonmyeloablative allogenic peripheral-blood stem cell transplantation. *N. Engl. J. Med.* **343,** 750–758.
51. Rini, B.L., Zimmermann, Y., Stadler, W.M., Gajeski, T.F., and Vogelzang, N.J. (2002) Allogenic stem-cell transplantation of renal cell cancer after nonmyeloablative chemotherapy: feasibility, engraftment and clinical results. *J. Clin. Oncol.* **20**, 2017–2024.
52. Bregni, M., Dodero, A., Peccatori, J., Pescarollo, A., Bernardi, M., Sassi, I., Voena, C., Zaniboni, A., Bordignon, C., and Corradini, P. (2002) Reduced-intensity haematopoietic cell allografting and escalating-dose donor lymphocyte infusions in advanced renal and breast cancer. *Blood* **99**, 4234–4236.
53. Pedrazzoli, P., Da Prada, G.A., Georgiani, G., Schiavo, R., Zambelli, A., Giraldi, E., Londonio, G., Locatelli, F., Siena, S., and Della Cuna, G.R. (2002) Allogenic blood stem cell transplantation after a reduced-intensity, preparative regimen: a pilot study in patients with refractory malignancies. *Cancer* **94**, 2409–2415.
54. Hentschke, P., Barkholt, L., Uzunel, M., Mattsson, J., Wersall, P., Pisa, P., Martola, J., Albiin, N., Wernerson, A., Soderberg, M., Remberger, M., Thorne, A., and Ringden, O. (2003) Low-intensity conditioning and haematopoietic stem cell transplantation in patients with renal and colon carcinoma. *Bone Marrow Transplant.* **31**, 253–261.
55. Ueno, N.T., Cheng, Y.C., Rondon, G., Tannir, N.M., Gajewski, J.L., Couriel, D.R., Hosing, C., de Lima, M.J., Anderlini, P., Khouri, I.F., Booser, D.J., Hortobagyi, G.N., Pagliaro, L.C., Jonasch, E., Giralt, S.A., and Champlin, R.E. (2003) Rapid induction of complete donor chimerism by the use of a reduced-intensity conditioning regimen composed of fludarabine and melphalan in allogenic stem cell transplantation for metastatic solid tumours. *Blood* **102,** 3829–3836.
56. Nakagawa, T., Kami, M., Hori, A., Kim, S.W., Murashige, N., Hamaki, T., Kishi, Y., Fujimoto, H., Matsuoka, N., Okajima, E., Komiyama, M., Tobisu, K., Wakayama, T., Uike, N., Tajima, K., Makimoto, A., Mori, S., Tanosaki, R., Takaue, Y., and Kakizoe, T. (2004) Allogenic haematopoietic stem cell transplantation with a reduced-intensity conditioning for treatment of metastatic renal cell carcinoma: single institution experience with a minimum 1-year follow-up. *Exp. Hematol.* **32**, 599–606.
57. Massenkeil, G., Roigas, J., Nagy, M., Wille, A., Stroszczynski, C., Mapara, M.Y., Loening, S., Dorken, B., and Arnold, R. (2004) Nonmyeloablative stem cell transplantation in metastatic renal cell carcinoma: delayed graft-versus-tumour effect is associated with chimerism conversion but transplantation has a high toxicity. *Bone Marrow*

*Transplant.* **34**, 309–316.

58. Blaise, D., Bay, J.O., Faucher, C., Michallet, M., Boiron, J.M., Choufi, B., Cahn, J.Y., Gratecos, N., Sotto, J.J., Francois, S., Fleury, J., Mohty, M., Chabamon, C., Bilger, K., Gravis, G., Viret, F., Braud, A.C., Bordou, V.J., Maraninchi, D., and Viens, P. (2004) Reduced-intensity preparative regimen and allogenic stem cell transplantation for advanced solid tumours *Blood* **103**, 435–441.
59. Tykodi, S.S., Warren, E.H., Thompson, J.A., Riddell, S.R., Childs, R.W., Otterud, B.E., Leppert, M.F., Storb, R., and Sandmaier, B.M. (2004) Allogenic haematopoietic stem cell transplantation for metastatic renal cell carcinoma after nonmyeloablative conditioning: toxicity, clinical response and immunological response to minor histocompatibility antigens. *Clin. Cancer Res.* **10**, 7799–7811.
60. Rini, B.I., Halabi, S., Barrier, R., Margdin, K.A., Avigon, D., Logan, T., Stadler, W.M., McCarthy, P.L., Lineker, C.A., Small, E.J., Cancer and Leukemia Group B, Eastern Cooperative Oncology Group, and Sothwestern Oncology Group (2006) Adoptive immunotherapy by allogenic stem cell transplantation for metastatic renal cell carcinoma: a CALGB Intergroup Phase II Study. *Biol. Blood Marrow Transplant.* **12,** 778–785.
61. Drachenberg, D. and Childs, R.W. (2003) Allogenic stem cell transplantation as immunotherapy for renal cell carcinoma: from immune enhancement to immune replacement. *Urol. Clin. North Am.* **30,** 611–622.
62. Bregni, M. et al. (2003) Graft versus tumour effect following reduced intensity allogenic stem cell transplantation in renal cell cancer: on behalf of the EBMT Solid Tumour Working Party. *Blood* **102,** 198.
63. Hojo, M., Morimoto, T., Maluccio, M., Asano, T., Morimoto, K., Lagman, M., Shimbo, T., and Suthanthiran, M. (1999) Cyclosporine induces cancer progression by a cell-autonomous mechanism. *Nature* **397**, 530–534.
64. Artz, A.S., Kocherginsky, M., and Van Besien, K. (2006) Order of patient entry influences outcome for metastatic renal cell cancer after non-myeloablative allogenic stem cell transplantation. *Br. J. Haematol.* **132**, 747–754.
65. Horowitz, M.M., Gale, R.P., Sandel, P.M., Goldman, J.M., Kersey, J., Kolb, H.J., Rimm, A.A., Ringden, O., Rozman, C., Speck, B., Truitt, R.L., Zwaan, F.E., and Bortin, M.M. (1990) Graft-versus-leukaemia reactions after bone marrow transplantation. *Blood* **75**, 555–562.
66. Gale, R.P., Horowitz, M.M., Ash, R.C., Champlin, R.E., Goldman, J.M., Rimm, A.A., Ringden, O., Stone, J.A., and Bortin, M.M. (1994) Identical twin bone marrow transplants for leukaemia. *Ann. Intern. Med.* **120,** 646–652.
67. Horowitz, M.M., Gale, R.P., Sondel, P.M., Goldman, J.M., Kersey, J., Kolb, H.J., Rimm, A.A., Ringden, O., Rozman, C., and Speck, B. (1990) Graft-versus-leukaemia reactions after bone marrow transplantation. *Blood* **75,** 555.
68 Hows, J., Bradley, B.A., Gore, S., Downie, J., Howard, M., and Gluckman, E. (1993) Prospective evaluation of unrelated donor bone marrow transplantation. *Bone Marrow Transplant.* **12**, 371–380.
69. Ringden, O., Schaffer, M., LeBlanc, K., Persson, U., Hauzenberger, D., Abedi, M.R., Olerup, O., Ljungman, P., and Remberger, M. (2004) Which donor should be chosen for haematopoietic stem cell transplantation among unrelated HLA-A, -B and –DRB1 genomically identical volunteers? *Biol. Blood Marrow Transplant.* **10,** 128–134.
70. Miyakoshi, S., Yuji, K., Kamo, M., Kusumi, E., Kishi, Y., Kobayashi, K., Murashige, N., Hamaki, T., Kim, S.W., Ueyama, J., Mori, S., Morinaga, S., Muto, Y., Masuo, S., Kanemaru, M., Hayashi, T., Takoue, Y., and Taniguchi, S. (2004) Successful engraftment after reduced-intensity umbilical cord blood transplantation for adult patients with advanced haematological disease. *Clin. Cancer Res.* **10**, 3586–3592.
71. Burgio, G.R., Gluckman, E., and Locatelli, F. (2003) Ethical reappraisal of 15 years of cord-blood transplantation. *Lancet* **361(9368),** 1566–1567.
